# Genetic Identification of Methicillin-Resistant *Staphylococcus aureus* Nasal Carriage and Its Antibiogram among Kidney Dialysis Patients at a Tertiary Care Hospital in AL-Karak, Jordan

**DOI:** 10.1155/2023/9217014

**Published:** 2023-03-15

**Authors:** Omar Al-Dmour, Rania Al-Groom, Ayman Alsheikh, Sameer Mahmoud, Kawther Amawi, Israa Yousef, Ayat Almaraira

**Affiliations:** ^1^Precision Medical Lab (PMLAB), AL-Karak, Jordan; ^2^Department of Allied Medical Sciences, Zarqa University College, Al-Balqa Applied University, Salt 2000, Jordan; ^3^Department of Medical Laboratory Science, Faculty of Allied Medical Sciences, Zarqa University, Zarqa 13110, Jordan; ^4^Department of Basic Medical Sciences, Faculty of Medicine, Al-Balqa Applied University, Al-Salt, Jordan; ^5^Department of Faculty of Science Requirement Unit, Faculty of Science, Zarqa University, Zarqa 13110, Jordan

## Abstract

**Background:**

Methicillin-resistant *Staphylococcus aureus* (MRSA) is a major bacterial pathogen.

**Aim:**

The present study aimed to determine the incidence of MRSA infections among kidney dialysis patients and the antibiotic susceptibility patterns and investigate the prevalence of mecA gene among MRSA isolates.

**Materials and Methods:**

A total of 83 nasal sterile cotton swabs samples were obtained from hemodialysis patients from Al-Karak Governmental Hospital, Al-Karak, Jordan. Collected and cultured on nutrient agar and mannitol salt agar and incubating at 37°C for 24–48 hours, *Staphylococcus aureus* (*S. aureus*) strains were identified by gram stain, coagulase test, and catalase tests. The MRSA isolates were tested for the presence of MecA and SCCmec genes using the Xpert SA Nasal Complete assay real-time PCR. Factors such as age and gender were included in the study. The antibiotic profile tested by using the disc diffusion method tested all MRSA isolates.

**Results:**

This study showed that 10.8% of the cultures' growth was *S. aureus* and 9.6% of all the patients were infected with MRSA, with no relationship between the number and frequency of MRSA according to the patient's gender or age. All MRSA (100%) isolates have both genes (MecA genes and SCCmec genes), and all samples were resistant to oxacillin, ceftazidime, cefoxitin, aztreonam, and ampicillin.

**Conclusion:**

The MRSA prevalence was determined among kidney dialysis patients in the hospital. All positive samples were resistant to oxacillin, ceftazidime, cefoxitin, aztreonam, and ampicillin, which is a very rare finding, and this will give the scientists and doctors a dangerous indication about health-care centers in the Al-Karak city of Jordan.

## 1. Introduction

Staphylococci are spherical-shaped bacteria that belong to the Micrococcaceae family and have a diameter of 0.5–1.8 m [1]. These bacteria are Gram-positive cocci, catalase-positive, oxidase negative, and they appear in microscopic clusters when stained [2]. Staphylococci are nonmotile, nonspore-forming bacteria that can withstand high salt concentrations and get their energy from aerobic respiration or fermentation [3]. Their nutritional needs are diverse, but they generally require an organic supply of nitrogen, as well as five to twelve important amino acids (such as arginine and valine) and B vitamins [4, 5].

*S. aureus* is a microorganism that is ubiquitous in both healthy and vulnerable hosts [6]. It can cause asymptomatic, long-term colonization of the human tissue or remain on the biofilm on the inert surface [7]. It can also cause fulminant invasion of the host with various symptoms [8, 9]. *S. aureus* can survive not only in human hosts but also in extreme environmental and animal conditions [10]. *S. aureus* developed resistance to methicillin immediately after the antibacterial drug was used clinically in 1961, and there has been a global pandemic of methicillin-resistant *Staphylococcus aureus* (MRSA) in both hospitals and community settings [11].

Their significant morbidity and mortality, as well as their resistance to all existing penicillins and most other beta-lactam medicines [12]. Penicillin-binding proteins (PBPs), which play an enzyme function in the formation of peptidoglycan, are present in the bacterial cell wall [13]. In contrast to MRSA, PBPs often have a low affinity for beta-lactam antibiotics, which leads to antibiotic resistance [14]. The mecA gene in MRSA encodes PBP2a, a PBP with limited antibiotic affinity [15],.and mecA, a structural gene on the *S. aureus* chromosome, identifies MRSA, and the femA and femB genes encode proteins that affect how resistant *S. aureus* is to methicillin [16].

Bacterial infections are one of the leading causes of death in hemodialysis patients [17]. In reality, hemodialysis patients are more susceptible to be infected because of their compromised immune systems and the nature of the hospital setting that are used in the dialysis session [18]. According to the recent reports, *S. aureus* is the most common bacterial infection in people who are receiving hemodialysis [19]. This infection can result from normal flora to more serious conditions like bacteremia, osteomyelitis, necrotizing pneumonitis, infective endocarditis, and toxic shock syndrome (TSS) [20].

Clinically [21], the major problem associated with *S. aureus* is the development of significant levels of resistance to multiple classes of antibiotics, making treatment difficult. Historically, *S. aureus* resistance emerged within two years of the introduction of penicillin [22].

*S. aureus* may cause a wide range of infections in humans [23]. The clinical infections of *S. aureus* are classified into community and nosocomial based on the origin of the infection [24].

However, the rate of *S. aureus* infections is rising. The bacterium can remain in a carrier state in the anterior nares for weeks or months without causing any infections [25]. When certain predisposing conditions, such as prolonged hospitalization, immune suppression, operations, the use of invasive medical equipment, and chronic metabolic illnesses occurs, the colonization progresses to infection [26]. Wound infections, infective endocarditis, osteoarticular infections, pleuropulmonary infections, and bacteremia are among the clinical infections caused by *S. aureus* [27]. Meningitis, urinary tract infections, and epidural abscesses are further clinical infections [28, 29]. This organism can enter the bloodstream and spread systemically to different organs causing sepsis. Food poisoning is caused by *S. aureus* releasing enterotoxins into the food, while toxic shock syndrome is caused by the release of superantigens into the bloodstream [30].

The aim of this study is as follows:

  To determine MRSA prevalence among kidney dialysis patients in Al-Karak Governmental Hospital
  To investigate antibiotics susceptibility patterns of MRSA by the disc diffusion method
  To detect mecA and SCCmec genes among MRSA isolates from kidney dialysis patients by Xpert SA Nasal Complete assay real-time PCR.

## 2. Materials and Methods

This cross-sectional study was conducted in Precision medical labs designed to determine the prevalence of MSSA and MRSA recovered from the nasal carriage of kidney dialysis patients and its antibiotic susceptibility pattern at AL-Karak, Jordan. According to the Jordan's Ministry of Health data for 2022, the overall population in the hemodialysis center at Al-Karak governmental hospital was involved in our study, with the prevalence of MRSA and MSSA among 83 hemodialysis patients at Al-Karak Governmental Hospital. Patients were prescreened by the treating physician for inclusion/exclusion in the study. Inclusion criteria included hemodialysis patients in Al-Karak Governmental Hospital, and exclusion criteria included patients who did not have a permanent residence in the dialysis department.

### 2.1. Samples Collection and Swab Processing

Nasal swabs were collected from 83 hemodialysis patients in Al-Karak Governmental Hospital with ages between 28- and 89 –years-old (35 males and 48 females). Data were collected over a period of 3 months between April 2022 and June 2022. All specimens collected by midturbinate nasal swabs from the hemodialysis patients using sterile dry cotton swabs were either inserted with the swab inside the nostril of the patients and rotate it and then put into the transport medium for later use in the culture or directly cultured on mannitol salt agar and then put in broth media and then processed by using the culture standardized method and incubated at 37C° for 24–48 Hrs. [31]. Swabs were grown on mannitol salt agar (MSA) either on the same day of collection or the day after for the initial presumptive isolation of *S. aureus*. To produce pure bacterial isolates, any yellow or yellow-orange colonies were selected and repeatedly subcultured on MSA. Only one colony from each subculture was chosen from the isolated colonies, and the inoculated plates were always incubated at 37°C for 48 hours.

### 2.2. Gram Stain Examination

A smear was prepared from the colonies in normal saline on a glass slide, dried, fixed, stained with the Gram's method, and examined under oil immersion lens.

### 2.3. Coagulase Test

To differentiate *Staphylococcus aureus* from other staphylococcus species, the coagulase test was performed on all isolates. Dense suspensions of Staphylococci from the culture are made on two ends of clean glass slide; one labeled as “test” and the other labeled as “control”. One drop of citrated plasma was added to the test suspension, and it was then thoroughly mixed.

### 2.4. Antibiotic Resistance of the Isolated *Staphylococcus aureus*

Antimicrobial susceptibility of the confirmed *S. aureus* isolates was determined first to methicillin using 1 *μ*g oxacillin discs by the disc diffusion method; in which discs were placed on the Muller–Hinton medium which permits the homogeneous diffusion of the antibiotics disc, and these plates swabbed with *S. aureus* were followed by incubation for 24 hours at 37°C according to the Clinical and Laboratory Standards Institute guidelines [31].

The principle of this method is as follows: It is to evaluate the sensitivity of *S. aureus* isolates with regards to the 24 antibiotics chosen for this study, commonly used for *S. aureus* treatment, as shown in Table 1; then, observing the development of the inhibition zone and then measuring the diameter of the inhibition zone, we finally decide on the *S. aureus* sensitivity and resistance to the antibiotics understudied.

### 2.5. Xpert® SA Nasal Complete

The GeneXpert instrument (GeneXpert® Dx System Version 5.1) system automates and integrates sample purification, nucleic acid amplification, and target sequence detection in simple or complex samples using real-time PCR and RT-PCR experiments. The systems require the employment of single-use, disposable cartridges that contain the reagents to host the PCR. Cross-contamination between the samples is prevented by the cartridges' self-contained design. The systems are made up of an instrument, a computer, and software that is already loaded for conducting tests and evaluating the findings.

The Xpert SA Nasal Complete assay was used according to the manufacturer's recommendations to comprise MRSA and SA detection assays in addition to a sample processing control (SPC) to ensure that the target bacteria were properly processed. The probe integrity, PCR tube filling in the cartridge, probe integrity, and dye stability were all verified by the probe check control (PCC).

The primers and probes in the Xpert SA Nasal Complete assay detected proprietary sequences for the staphylococcal protein A (spa), the gene for methicillin/oxacillin resistance (mecA), and the staphylococcal cassette chromosome (SCCmec) inserted into the SA chromosomal attB site. The primers used are shown in Table 2.

### 2.6. Procedure

- After swabs were taken from bacteria colonies on the surface of the MSA agar plates, the swabs were placed into the plastic transport tube and moistened with 2–3 drops of sterile physiological saline.
- Swabs were inserted all the way inside the tube until they rested on top of the sponge at the bottom. Tubes were labeled with the patient's ID, kept at room temperature (15–30°C), and transported to the GeneXpert test site. All experiments were done within one hour after placing the swab in the tubes.
- Cartridges and elution reagent were removed from the packages, and then the swabs were inserted into the elution reagent tubes and were broken down.
- The elution vial covers were closed and shaken by a vortex for 10 seconds at high speed.
- The cartridges cover was removed. Then, using a sterile transfer pipette, all elution reagent contents were transferred to the “S” chamber of the Xpert SA Nasal Complete assay cartridge and cartridges covers were closed. The tests were started within 15 minutes of loading the sample into the cartridge.
- The GeneXpert instrument system software was logged in, and the data were automatically interpreted based on fluorescence signals and incorporated computation algorithms.
- *S. aureus* subsp. aureus ATCC 25923 and *S. aureus* subsp. aureus ATCC 25923 were used as positive controls. Methicillin-sensitive*Staphylococcus* epidermidis ATCC 1228 was used as the negative control.
- For positive MRSA results, all MRSA targets (spa, mecA, and SCCmec) had a Ct within the valid range and endpoint above the threshold setting. For Negative MRSA results, target DNA for mecA was not detected and target DNA for SCCmec was also not detected.

### 2.7. Statistical Analysis of Data

All analysis were performed by using Statistical Package for Social Sciences (SPSS), version 22.0 (IBM Corporation, Armonk, NY). Percentage and frequencies were used for the categorical variables. Pearson's correlation test was used for the association between the continuous variables. Moreover, the one-way ANOVA test was used to explore the samples' demographics.

## 3. Results and Discussion

### 3.1. *S. aureus* Detection

#### 3.1.1. Gram Stain Examination

*S. aureus* organisms were seen as Gram-positive cocci, approximately 1 *μ*m in diameter, usually arranged in clusters like grape.

### 3.2. Coagulase Test

When the cocci clump together or agglutinate within 5 to 10 seconds, the strain was considered positive for *S. aureus*.

### 3.3. Antimicrobial Susceptibility Testing

 All MRSA targets (spa, mecA, and SCCmec) had a Ct within the valid range and endpoint above the threshold setting. For negative MRSA results, target DNA for mecA was not detected and target DNA for SCCmec was also not detected.

### 3.4. MRSA Detection

**Figure 1:**
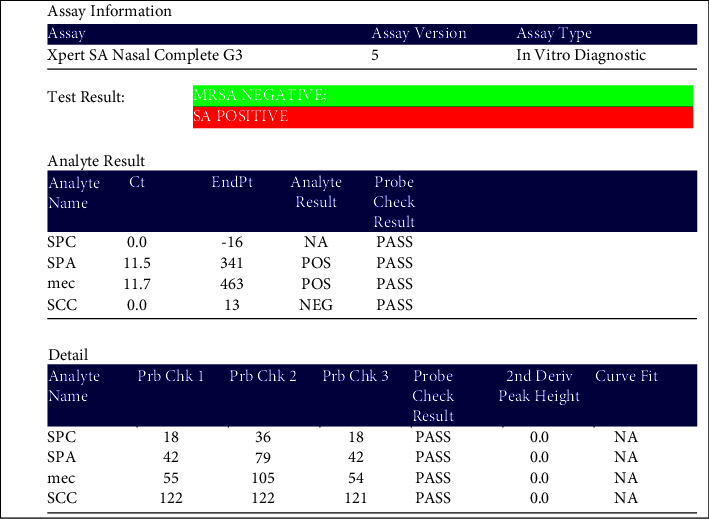
Xpert SA nasal complete G3 of the (mecA) and (SCCmec) MRSA negative result.

**Figure 2:**
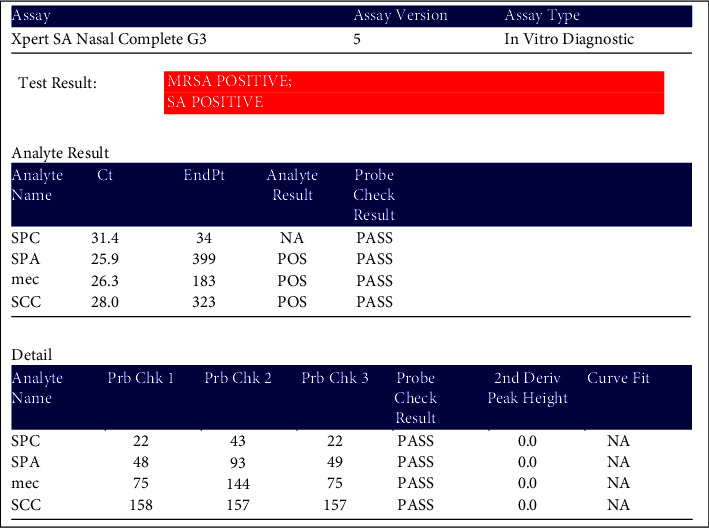
Xpert SA nasal complete G3 of the (mecA) and (SCCmec) MRSA positive result.

The primers and probes in the Xpert SA Nasal Complete assay real-time PCR and RT-PCR detected proprietary sequences for the staphylococcal protein A (spa), the gene for methicillin/oxacillin resistance (mecA), and the staphylococcal cassette chromosome (SCCmec) inserted into the *S. aureus* chromosome alatt B site. Primers associated with the specific genes were mentioned in the methodology chapter. The examples of the results are shown in Figures 1 and 2.

Methicillin-resistant *Staphylococcus aureus* (MRSA) was detected using Xpert real-time PCR, and the results are shown in Table 3, which shows that there were 8 MRSA out of 83 patients, which consists of 9.6%.

### 3.5. The Prevalence of MRSA

Dialysis patients are highly susceptible to infections, frequently those caused by antimicrobial-resistant organisms, including MRSA [32]. In hemodialysis patients, the vascular access site in infections is closely associated to *S. aureus* bacteremia (SAB) [33]. Understanding and evaluating the bacterial infection sources, risk factors associated with it, and how the bacteria transmission occurs will be helpful in the planning to prevent and control the infections. Prevalence studies of MRSA or any bacterial infection will be of great help for the prevention required in hospital wards to save patients and their lives. Because methicillin-resistant*S. aureus* is one of the most important causes of nosocomial infections worldwide and usually acquired via spreading, one of the most effective methods for preventing the spread of MRSA requires the detection of colonized patients and healthcare workers and the assessment of risk factors associated with colonization.

To recognize *S. aureus* bacteria from the other bacterial species, catalase and coagulase tests were used in the present study, according to the following references [34, 35].

In the current study, we found that we have around 10.8 percent of the samples and the size of it is SA = 9 and *n* = 83.

The MRSA-specific genes which are enable to recognize the strain of methicillin-resistant *Staphylococcus aureus* were used in the current study, namely, the gene for methicillin/oxacillin resistance (mecA) and the staphylococcal cassette chromosome (SCCmec). Many studies used these genes to recognize the MRSA. The primers and probes in the Xpert SA Nasal Complete assay real-time PCR and RT-PCR enabled to detect specific sequences for the staphylococcal protein A (spa), the gene for methicillin/oxacillin resistance (mecA), and the staphylococcal cassette chromosome (SCCmec) inserted into the SA chromosomal attB site [36–39].

In the current study, eight of the 83 tested samples were found to contain MRSA, while 54 were negative, nine contained *Staphylococcus aureus,* and 20 corresponds to growth out. Hence, the global sample (*N* = 83) is composed of 9.6% of MRSA, 10.84% of SA, and 24.1% for growth out. These findings are consistent with the previous studies with difference in the region and *S. aureus* is a prominent human and animal opportunistic pathogen. Both methicillin-sensitive*S. aureus* (MSSA) and methicillin-resistant*S. aureus* (MRSA) can cause mild to fatal diseases, spread locally and internationally, colonize a variety of human body areas, and survive outside of the hosts [40]. David and Daum in 2010 found that the majority of invasive MRSA infections can be linked to a hospital stay or other health-care exposure, and 15% of invasive infections occur in people who have never been exposed to health care. Recent studies suggest that the MRSA community was associated with skin infections, such as abscesses, boils, and other pus-filled lesions in the hospital wards, with some differences between the countries [41, 42].

*S. aureus* is Gram-positive coccoid bacteria of approximately 1.5 *μ*m in diameter that is extensively disseminated in nature [43], and *S. aureus* strains are susceptible to furazolidone (100 *μ*g) and are resistant to minimum levels of bacitracin (0.04 units) [44]. They are susceptible to lysis by lysostaphin and are relatively resistant to lysis by lysozyme and are found in humans [45]. The most virulent include *S. aureus* and *S. lugdudensis* in humans and *S. aureus* and *S. intermedius*, although the predominant mechanism of *S. aureus* transmission is direct contact, typically the skin-to-skin contact with a colonized or infected individual [46]. One of the most important biological properties of *Staphylococcus* is its ability to colonize healthy humans asymptomatically [47]. In this study, approximately 10.8 percent of the tested samples contained *Staphylococcus aureus* (SA) (SA = 9, *n* = 83). The main objective of this study is to determine the prevalence of MRSA nasal carriage among patients in the dialysis department at Al-Karak Governmental Hospital. Results demonstrated clearly a low prevalence rate of *S. aureus* nasal carriage (10.8%) and also a low prevalence of MRSA nasal carriage (9.6%). These rates are lower than those reported for other areas of the world. A study on the MRSA prevalence in Portugal's hospitals, using nasal carriage among the patients, reported a very low prevalence of this nasal carriage in the examined patients, corresponding to 4.8% [48].

Another study conducted at the King Fahd Hospital and a Tertiary Care Canteen in the eastern province of Saudi Arabia involved 205 patients with kidney disease. Results showed the presence of *S. aureus* in 38% of the samples, of which only 10.7% were MRSA, with a high incidence in the age group of 75–84 years. [41].

In 2014, Hassoun conducted a study on the prevalence of MRSA in Europe and the results were as follows. The percentage of invasive MRSA isolates in Netherlands was 0.9% while 56% in Romania. MRSA prevalence exhibits a north-south variation in Europe, with a higher proportion of resistant isolates in southern countries compared with northern countries [49].

Prevalence of MRSA in veterinary personnel ranged from 0 to 50%, with an overall average prevalence of 8% for all studies, and the prevalence was less than 10% in most MRS studies with an overall mean prevalence of 8% across the studies; the prevalence was less than 10% in most studies done on the MRSA [11].

A cross-sectional study conducted by Peters et al. [50] in Hamburg showed that the number of MRSA colonization's among the nursing staff and residents of geriatric nursing homes ranged between 1.6% and 5.5%.

Peacocks et al. recorded that the prevalence of nasal carriage of *S. aureus* is between 20% and 25% of the hospital sitting, and the transient colonization of *S. aureus* affect at least 60% of the residual population [51].

### 3.6. ANOVA Statistical Analysis

One-way ANOVA tests were done to check the significance of the variables on the patients, and Tables 4 and 5 show the gender and age ANOVA results. This shows that there is no significant relationship neither with gender nor with age at the respective *P* values of (*P*=0.694) and (*P*=0.718).

### 3.7. The Association between the Age and Gender with the MRSA and SA

The female group was the dominant group in the study with 57.8 percentage comparing with males (42.2%), which gave us the normal percentage in the normal populations, which contains females higher than males. Age was categorized under groups: the most important sample corresponds to the age group of 70–79 years, which also contains the highest rate of females. These findings confirm the results published in a previous study performed in Saudi Arabia. Indeed, in a Saudi Arabia hemodialysis center, the prevalence of MRSA was 38% (58.7% among the 75–84 year's age group and 50% in the 65–74 year's age group) [41].

Regarding the MRSA and SA infection, the highest group of infection was 60–69 -years-old. Female infection rates were higher than males according to the previous chapter tables.

In 2017, a study done in Malaysia showed that the male gender and patient >50 years of age (*P* < 0.0001) were significantly associated with the increased risk of MRSA acquisition, which disagrees with our results and this may be due to many reasons as we will mention later in the limitations such as populations differences and samples [52].

Gorey et al. performed a study on the MRSA prevalence, and their results were in disagreement with our results concerning age as patients <18 -years-old were more likely to be colonized by *S. aureus* compared to the patients above 61 years. While their results were consistent with our results regarding the proportion of MRSA among isolates which was 59 (72%) [53].

### 3.8. The Correlation Studies

The correlation study was done between all the variables, and the correlation showed that there is an association between the MRSA and age variable with a significance value (*P*=0.021). Indeed, there is correlation between the age and growth of SA and MRSA, which showed that the age decreasing will decrease the incidence of infection of growth, SA infection in general and specially in MRSA. Nonetheless, there is a negative correlation between the gender and the growth of' SA and MRSA which showed that the probability to be male is correlated with the low incidence of the infection in general, especially with MRSA. Tables 6 and 7 show the correlation study result.

### 3.9. Antibiotic Statistical Analysis

Statistical analysis of antibiotic sensitivity/resistance was done on the frequencies. Thus, for such analysis, we changed the symbols as follows: (0 = R, 1 = Inter, 2 = S); for every sample, the SD will be near 1; if it is above, this means that the sensitivity to antibiotic is more important. If the *M* and SD are less than 1, this means that the sample is more sensitive to the antibiotic.  Table 8 shows the antibiotic statistical analysis.

Samples antibiotic frequencies are as follows for every sample in Tables 9 and 10.

### 3.10. Antibiotic Disc Resistance and Sensitivity Diameters

The sensitivity, resistance, and intermediate sensitivity are the categories of the antibiotic sensitivity for all antibiotics, and according to the standards which are shown in the methodology chapter, the results were categorized.  Table 11 shows the antibiotic sensitivity diameters that we found, antibiotic disc resistance, and sensitivity.

Results of antibiotic resistance and sensitivity are different from patient to patient, depending on the bacteria strain and then on the genetics composition and whether the antibiotic is mistakenly or irrationally taken. Antibiotic resistance comes from the misuse of the antibiotic, which let the bacteria develop antibiotic resistance to the target antibiotic. Shaki's study in India in 2014 found that only 1% of their isolates had a sensitive reaction to ciprofloxacin and penicillin and 46% were sensitive to tetracycline. While in our study, 22.2% of our isolates are sensitive to ciprofloxacin, which is not agreeing with the previous findings, but these sensitivity tests depend on many reasons that were explained well. They also found that the isolates were sensitive to ceftriaxone and spectinomycin, which can therefore be used as the first-line drug for syndromic management of urethritis, but in our samples, some of the sare sensitive and some others are resistant as shown in Table 12 [54].

Interestingly, we found that all bacterial strains isolated from the studied samples were resistant to oxacillin, ceftazidime, cefoxitin, aztreonam, and ampicillin, which is a rare finding and this will give the scientists and doctors a dangerous indication about health-care centers in the Al-Karak city of Jordan. There are many reasons for the formation of antibiotic-resistant strains; in particular, the misuse of Community medicines which can cause antibiotic resistance due to taking intermittent doses of antibiotics.

The results of Vestergaard and others' study results agree with our antibiotic resistance results. *S. aureus* is considered the resistant bacteria to all classes of antibiotics clinically available, and resistance can develop mutations in chromosomal genes or through the acquisition of horizontally transferred resistance determinants [55].

*S. aureus* is generally benign, but antibiotic resistance contributes to the success of *S. aureus* as a human pathogen [56].

The results also show that there are no isolated strains from the tested samples which are sensitive to all antibiotics, which indicates that in the dialysis department, the nurses and doctors cannot give a general antibiotic to all the patients. Bacterial culture will be compulsory for all the patients with infection to check which type of antibiotic is the strain. This will cause more costs and delay in the treatment according to the culture time consuming (3 days).

## 4. Conclusion

The MRSA prevalence was determined among kidney dialysis patients in Al-Karak Hospital. The results showed that there were 8 cases contaminated by MRSA out of 83 patients, which consists of 9.6%, which agrees with some studies and disagree with others according to the prevalence rates found in each one.

The antibiotic sensitivity and resistance were measured among the positive growth of kidney dialysis patients in Al-Karak Hospital. The results found that all the bacteria isolated from the samples which is resistant to the tested antibiotics (oxacillin, ceftazidime, cefoxitin, aztreonam, and ampicillin), which is a very rare finding that will give the scientists and doctors a dangerous indication about health-care centers in the Al-Karak city of Jordan. Many reasons of the antibiotic resistance strains formation could include the community overuse of antibiotics, which can induce strains resistance to antibiotics [57, 58].

## 5. Recommendations

- Personal protective equipment is used, especially masks, by all staff working with the patients when they are in direct contact.
- There is a need for a widespread screening program for MRSA nasal carriage in all hospitals to know the exact prevalence of MRSA nasal carriage in Jordan.
- There is a need for a screening program for antibiotic-resistant microorganisms in our hospitals.
- Rational antibiotic prescribing based on local guidelines to prevent the development of bacterial resistance.

## Figures and Tables

**Table 1:** *S. aureus* isolates tested for susceptibility to antibiotics.

Antibiotics	Symbol	MIC	Concentration (*μ*g)
Ampicillin	AM	29	10
Aztreonam	ATM	22	30
Amikacin	AK	17	30
Cefotaxime	CTX	23	30
Ceftriaxone	CRO	21	30
Cefuroxime	CXM	18	30
Cefoxitin	FOX	22	30
Ciprofloxacin	CIP	21	5
Cotrimoxazole	SXT	16	25
Gentamicin	CN	15	10
Imipenem	IPM	16	10
Levofloxacin	LEF	19	5
Nitrofurantoin	F	17	300
Norfloxacin	NOR	17	10
Tazocin	TPZ	16	
Cephalothin	KF	18	30
Cefixime (suprax)	CFM	19	5
Ertapenem	ETP	19	10
Pipercallin	PRL	21	100
Amoxicillin + clavulanic acid	AMC	20	30
Ceftazidime	CAZ	18	30
Meropenem	MEM	16	10
Cefazolin	CZ	18	30
Oxacillin	OX	13	1

**Table 2:** Primer sets used to characterize methicillin-resistant*Staphylococcus aureus*.

Loci	Primer sequences (5′ 3′)
MecA	F: GGCATCGTTCCAAAGAATGT
	R: CCATCTTCATGTTGGAGCTTT

SCCmec	F: CATTTGTGAAACACAGTACG
	R: GTTATTGAGACTCCTAAAGC

**Table 3:** MRSA frequencies in the current study.

MRSA		Frequency	Percent	Valid percent	Cumulative percentage
Valid	Neg	75	90.4	90.4	90.4
	Pos	8	9.6	9.6	100.0
	Total	83	100.0	100.0	

**Table 4:** Gender ANOVA.

Gender	Sum of squares	df	Mean square	*F*	Sig.
Between groups	0.769	5	0.154	0.608	0.694
Within groups	19.472	77	0.253		

Total	20.241	82			

**Table 5:** Age ANOVA.

Age	Sum of squares	df	Mean square	*F*	Sig.
Between groups	0.217	1	0.217	0.132	0.718
Within groups	133.421	81	1.647		

Total	133.639	82			

**Table 6:** Bivariate statistical analysis, dependent variable: age.

Source	Type III sum of squares	Standard df	Mean square	*F*	Sig.
Corrected model	5.825^a^	6	0.971	0.577	0.747
Gender	0.079	1	0.079	0.047	0.829
Growth	0.263	1	0.263	0.156	0.694
Staph	4.018	1	4.018	2.389	0.126
MRSA	2.381	1	2.381	1.416	0.238
Gender *∗* Growth	1.432	1	1.432	0.851	0.359
Error	127.814	76	1.682		
Total	1576.000	83			
Corrected total	133.639	82			

a. *R* squared = 0.044 (adjusted *R* squared = −0.032).

**Table 7:** Gender bivariate statistical analysis, dependent variable: gender.

Source	Type III sum of squares	df	Mean square	*F*	Sig.
Corrected model	2.013^a^	13	0.155	0.586	0.857
Intercept	1.358	1	1.358	5.139	0.027
Growth	0.131	1	0.131	0.495	0.484
Age	1.025	5	0.205	0.776	0.570
Growth *∗* Age	0.245	3	0.082	0.309	0.819
Error	18.228	69	0.264		
Total	35.000	83			
Corrected total	20.241	82			

a. *R* squared = 0.099 (adjusted *R* squared = −0.070)

**Table 8:** The antibiotic statistical analysis.

		S7	S11	S15	S19	S20	S33	S53	S76	S80
*N*	Valid	24	24	24	24	23	24	24	24	24
	Missing	0	0	0	0	1	0	0	0	0
Mean		0.75	0.33	0.79	0.71	0.96	0.50	0.67	0.92	0.79
Std. deviation		0.989	0.761	0.932	0.955	1.022	0.885	0.963	1.018	0.977
Variance		0.978	0.580	0.868	0.911	1.043	0.783	0.928	1.036	0.955
Range		2	2	2	2	2	2	2	2	2

**Table 9:** Sample 20 antibiotic.

		Frequency	Percent	Valid percent	Cumulative percent
Valid	R	12	50.0	52.2	52.2
	S	11	45.8	47.8	100.0
	Total	23	95.8	100.0	
Missing	System	1	4.2		

Total		24	100.0		

**Table 10:** S33.

		Frequency	Percent	Valid percent	Cumulative percent
Valid	R	18	75.0	75.0	75.0
	S	6	25.0	25.0	100.0
	Total	24	100.0	100.0	

**Table 11:** The antibiotic sensitivity diameters per (mm).

Antibiotics	Symbol	S7	S11	S15	S19	S20	S33	S53	S76	S80
Ampicillin	AM	17	15	15	19	10	11	16	22	21
Aztreonam	ATM	15	12	12	6	11	10	11	14	15
Amikacin	AK	17	13	14	19	10	19	7	20	18
Cefotaxime	CTX	9	11	24	23	23	14	25	23	26
Ceftriaxone	CRO	25	8	21	12	13	13	11	10	15
Cefuroxime	CXM	18	13	21	19	23	13	14	18	20
Cefoxitin	FOX	11	5	17	21	13	21	15	17	15
Ciprofloxacin	CIP	15	23	11	14	25	9	11	7	14
Cotrimoxazole	SXT	3	10	19	8	16	17	20	16	5
Gentamicin	CN	19	12	13	10	15	11	18	15	7
Imipenem	IPM	21	9	17	23	16	25	20	17	19
Levofloxacin	LEV	11	15	15	23	22	14	15	12	13
Nitrofurantoin	F	10	7	15	17	19	24	11	14	18
Norfloxacin	NOR	12	17	11	19	9	11	11	10	12
Taconic	TPZ	24	19	15	17	11	15	9	20	13
Cephalothin	KF	21	11	19	13	23	14	14	18	21
Cefixime suprax	CFM	9	19	11	15	14	15	15	19	14
Ertapenem	ETP	10	14	17	18	24	13	21	19	19
Piperacillin	PRL	15	9	17	17	16	21	22	25	10
Amoxicillin clavulanic acid	AMC	24	17	19	14	21	18	11	19	15
Ceftazidime	CAZ	8	6	5	3	13	14	14	11	10
Meropenem	MEM	19	13	17	16	21	19	21	11	16
Cefazolin	CZ	10	11	23	14	5	3	18	6	22
Oxacillin	OX	10	2	5	2	9	10	7	9	3

**Table 12:** Antibiotics resistance and sensitivity tests.

Antibiotics	Symbol	7	11	15	19	20	33	53	76	80
Ampicillin	AM	R	R	R	R	R	R	R	R	R
Aztreonam	ATM	R	R	R	R	R	R	R	R	R
Amikacin	AK	S	R	R	S	R	S	R	S	S
Cefotaxime	CTX	R	R	S	S	S	R	S	S	S
Ceftriaxone	CRO	S	R	S	R	R	R	R	R	Inter
Cefuroxime	CXM	S	R	S	S	S	R	R	S	S
Cefoxitin	FOX	R	R	R	R	R	R	R	R	R
Ciprofloxacin	CIP	R	S	R	R	S	R	R	R	R
Cotrimoxazole	SXT	R	R	S	R	S	S	S	S	R
Gentamicin	CN	S	R	R	R	S	R	S	S	R
Imipenem	IPM	S	R	S	S	S	S	S	S	S
Levofloxacin	LEF	R	R	R	S	S	R	R	R	R
Nitrofurantoin	F	R	R	Inter	S	S	S	R	R	S
Norfloxacin	NOR	R	S	R	S	R	R	R	R	R
Tazocin	TPZ	S	S	Inter	R	R	R	R	S	R
Cephalothin	KF	S	R	S	R	S	R	R	S	S
Cefixime (suprax)	CFM	R	S	R	R	R	R	R	S	R
Ertapenem	ETP	R	R	Inter	Inter	S	R	S	S	S
Piperacillin	PRL	R	R	R	R	R	S	S	S	R
Amoxicillin clavulanic acid	AMC	S	R	R	R		R	R	R	R
Ceftazidime	CAZ	R	R	R	R	R	R	R	R	R
Meropenem	MEM	S	R	S	S	S	S	S	R	S
Cefazolin	CZ	R	R	S	R	R	R	S	R	S
Oxacillin	OX	R	R	R	R	R	R	R	R	R

## Data Availability

The database analyzed for the current study is available from the corresponding author upon reasonable request.
